# Reproductive and hormonal risk factors for sarcoidosis: a nested case–control study

**DOI:** 10.1186/s12890-022-01834-1

**Published:** 2022-01-24

**Authors:** Marina Dehara, Michael C. Sachs, Susanna Kullberg, Johan Grunewald, Anders Blomberg, Elizabeth V. Arkema

**Affiliations:** 1 Clinical Epidemiology Division, Department of Medicine Solna, Karolinska Institutet, Stockholm, Sweden; 2 Department of Medical Epidemiology and Biostatistics, Karolinska Institutet, Stockholm, Sweden; 3 Respiratory Medicine Division, Department of Medicine Solna, Karolinska Institutet, Stockholm, Sweden; 4 Center for Molecular Medicine, Karolinska Institutet and Karolinska University Hospital, Stockholm, Sweden; 5 Respiratory Medicine, Theme Inflammation and Ageing, Karolinska University Hospital, Stockholm, Sweden; 6 Department of Public Health and Clinical Medicine, Section of Medicine, Umeå University, Umeå, Sweden

**Keywords:** Female hormones, Nested case–control studies, Reproduction, Risk factors, Sarcoidosis, Women

## Abstract

**Background:**

Sarcoidosis incidence peaks in females around the fifth decade of life, which coincides with menopause, suggesting hormonal factors play a role in disease development. We investigated whether longer exposure to reproductive and hormonal factors is associated with reduced sarcoidosis risk.

**Methods:**

We conducted a matched case–control study nested within the Mammography Screening Project. Incident sarcoidosis cases were identified via medical records and matched to controls on birth and questionnaire date (1:4). Information on hormonal factors was obtained through questionnaires prior to sarcoidosis diagnosis. Multilevel modelling was used to estimate adjusted odds ratios with 95% credible intervals (OR; 95% CI).

**Results:**

In total, 32 sarcoidosis cases and 124 controls were included. Higher sarcoidosis odds were associated with older age at menarche (OR 1.19: 95% CI 0.92–1.55), natural menopause versus non-natural (OR 1.53: 95% CI 0.80–2.93), later age at first pregnancy (OR 1.11: 95% CI 0.76–1.63) and ever hormone replacement therapy (HRT) use (OR 1.40: 95% CI 0.76–2.59). Lower odds were associated with older age at menopause (OR 0.90: 95% CI 0.52–1.55), longer duration of oral contraceptive use (OR 0.70: 95% CI 0.45–1.07), longer duration of HRT use (OR 0.61: 95% CI 0.22–1.70), ever local estrogen therapy (LET) use (OR 0.83: 95% CI 0.34–2.04) and longer duration of LET use (OR 0.78: 95% CI 0.21–2.81). However, the CIs could not rule out null associations.

**Conclusion:**

Given the inconsistency and modest magnitude in our estimates, and that the 95% credible intervals included one, it still remains unclear whether longer estrogen exposure is associated with reduced sarcoidosis risk.

**Supplementary Information:**

The online version contains supplementary material available at 10.1186/s12890-022-01834-1.

## Background

Sarcoidosis is a multisystem inflammatory disease of hitherto unknown etiology which mainly affects the lungs and lymph nodes [1]. In Sweden, 1100 new cases are diagnosed every year in adults, with a peak incidence in males between ages 30–50 years, and in females between 50 and 60 years [2]. These two different peaks suggest that sex-specific factors play a role in disease onset. Endogenous hormones, especially estrogens, may delay sarcoidosis onset and reduce its severity in women through resetting the imbalanced T-helper (Th)1/Th2 immune response [3]. Estrogen exposure during a woman’s lifetime is highly related to several reproductive factors, including age at menarche, pregnancy, menopause (proxies of endogenous hormones) or use of exogenous hormones such as oral contraceptives [(OC) progestin-only, or combined estrogen–progestin formulations] and hormone replacement therapy [(HRT) estrogen-only, or combined estrogen-progesterone formulations].

Some reports indicate that patients with sarcoidosis often undergo remission during pregnancy, suggesting a favorable effect of endogenous hormones [4, 5]. However, these studies concerned the clinical course of sarcoidosis among women already diagnosed with the disease. We are aware of only one epidemiological study that has investigated the association between reproductive and hormonal factors and risk of incident sarcoidosis [6]. A higher age at menopause, later age at first birth and having a more recent birth (indicators of longer exposure to estrogen) were associated with a lower risk of sarcoidosis in black women from the United States [6]. A protective effect of estrogen on sarcoidosis may explain the observation that women are more often diagnosed with sarcoidosis after 50 years of age [2] around the time of menopause, when estrogen levels decrease dramatically [7]. However, further investigation is needed to determine if estrogens are etiologically linked to sarcoidosis.

We performed a nested case–control study using information from the Mammography Screening Project (MSP). Our aim was to investigate whether longer exposure to estrogen, proxied by reproductive and hormonal factors, is associated with subsequent reduced risk of sarcoidosis in women.

## Methods

### Study population

We used a matched case–control study design with cases and controls selected from the MSP, which has been presented in detail elsewhere [8]. Briefly, women aged 18–82 years (95% were 48–70 years old) living in Västerbotten County were invited to undergo mammography every 2–3 years between 1995 and 2006. During the mammography screening visit, women completed a self-administered questionnaire assessing reproductive conditions (e.g. age at menarche, parity, age at menopause), use of OC and HRT, use of other types of medications, smoking habits, and self-reported weight and height. The MSP questionnaire is provided in the Additional file 1: Mammography survey.  Some women attended the mammography screenings more than once and, thus, have completed the MSP questionnaire multiple times. Eighty-five percent of all invited women attended the mammography screening and were included in the MSP.

### Identification of cases and controls

Incident sarcoidosis cases were identified who received an International Classification of Diseases (ICD)-9 135 or ICD-10 D86 diagnosis at the University Hospital of Umeå [9]. Sarcoidosis diagnosis was validated via thorough review of medical records and classified as pulmonary or non-pulmonary sarcoidosis. We identified 46 cases who participated in the MSP. Four were excluded because the diagnosis could not be confirmed via medical record review and 10 cases were excluded because they had been diagnosed before the questionnaire date. We were left with 32 cases for analysis. Controls without a diagnosis of sarcoidosis were selected from the MSP population in a ratio of 4 to 1, matched on birthdate (± 6 months) and questionnaire date (± 3 months).

### Exposures: reproductive and hormonal factors

Information on reproductive and hormonal factors was obtained from the MSP questionnaires at the time of recruitment in the cohort or during follow-up questionnaires prior to sarcoidosis diagnosis. A detailed description of the exposure variables is presented in Table 1.

**Table 1:** Description of variables used to investigate reproductive and hormonal factors obtained from the Mammography Screening Project

Variable	Type	Comment
*Proxies of endogenous estrogen exposure*		
Age at menarche, years	Continuous	Based on the question: “Age at menarche?”
Total menstrual lifespan, years	Continuous	Calculated as age at menopause (or age at questionnaire for pre- or peri-menopausal women) minus age at menarche with additional subtraction of 1 year for each pregnancy (number of pregnancies × 1 year) and duration of OC use [10, 11].
Age at menopause, years	Continuous	Based on the question: “Age when reached menopause?”
Menopausal status	Categorical: pre- or peri-menopausal; post-menopausal; unknown menopausal status	*Pre-menopausal* Women who were < 45 years of age and/or whose menstrual periods had not ceased for more than 6 months (apart from pregnancy and breastfeeding) were considered pre-menopausal. *Peri-menopausal* Women who had menstruated within the last 12 months, were taking HRT, and were > 45 years of age were considered peri-menopausal. *Post-menopausal* Women who were taking HRT and had not undergone a hysterectomy, and/or whose menstrual periods had ceased for at least 12 months, or who were > 60 years of age were considered post-menopausal.
Natural menopause	Binary: no; yes	Women who answered YES to the question: “Menstruation ceased naturally?” were considered as naturally menopausal. Women who answered YES to the questions: “Menstruation ceased due to ovariectomy?” and/or “Menstruation ceased due to hysterectomy?” were considered as non-naturally menopausal.
*Pregnancy-related factors*		
Ever pregnant	Binary: nulligravid; gravid	Defined from the number of pregnancies variable. *Nulligravid* Women who reported 0 pregnancies. *Gravid* Women who reported 1 or more pregnancies.
Number of pregnancies	Continuous	Based on the question: “Number of pregnancies?”
Age at first pregnancy, years	Continuous	Based on the question: “Year of first pregnancy?” Calculated as year of first pregnancy minus year of birth.
Age at last pregnancy, years	Continuous	Based on the question: “Year of last pregnancy?” Calculated as year of last pregnancy minus year of birth.
Years since last pregnancy	Continuous	Calculated as the difference between age at questionnaire and age at last pregnancy.
*Proxies of exogenous estrogen exposure*		
OC use	Binary: never; ever	Based on the question: “Have you ever used oral contraceptives?” OCs include progestin-only or combined estrogen–progestin formulations. Information on formulation type was not collected on the MSP questionnaire.
Duration of OC use, years	Continuous	Based on the question: “For how long have you been using oral contraceptives?”
Age at first OC use, years	Continuous	Based on the question: “How old were you the first time you used oral contraceptives?”
HRT use	Binary: never; ever	Based on the question: “Are you currently using or have you in the past been using hormone replacement therapy for menopausal symptoms?” HRT includes estrogen-only or combined estrogen–progesterone formulations administered orally. Information on formulation type was not collected on the MSP questionnaire.
Duration of HRT use, years	Continuous	Based on the question: “For how long, in total, have you been using hormones for menopausal symptoms?”
Age at first HRT use, years	Continuous	Based on the question: “How old were you the first time you used hormones for menopausal symptoms?”
LET use	Binary: never; ever	Based on the question: “Have you been using estrogen (tablets or locally) for dry mucous membranes?” LET is a form of HRT that corresponds to the local/vaginal administration in the form of estrogenic creams and vaginal tablets.
Duration of LET use, years	Continuous	Based on the question: “For how long have you been using estrogen (tablets or locally) for dry mucous membranes?”

*OC* oral contraceptives, *HRT* hormone replacement therapy, *LET* local estrogen therapy, *MSP* Mammography Screening Project

### Other variables

Data on body mass index (BMI) and smoking were obtained from the MSP questionnaires at the time of recruitment in the cohort (if missing, follow-up questionnaires were used). BMI was calculated as weight in kilograms divided by height in meters squared and kept as a continuous variable in the analysis. Smoking status was categorized into non-smoker vs. smoker. These variables are potential confounders since previous studies have found these factors to be associated with both sarcoidosis [12–15] and women’s sex hormones [16, 17].

### Statistical analysis

Characteristics of sarcoidosis cases and controls from the MSP were reported as means with standard deviations, as medians with ranges or as proportions. Bayesian hierarchical regression analysis, also known as multilevel modelling, by data augmentation was used to estimate adjusted odds ratios with 95% credible intervals (OR; 95% CI) for the associations between multiple reproductive and hormonal factors and incident sarcoidosis. The OR was used to estimate the risk ratio. The Bayesian 95% credible intervals are interpreted as the probability that the true (unknown) odds ratio would lie within the interval is equal to 0.95, given the evidence provided by the observed data [18]. The point estimate (the median) is the most probable value, while values in the extreme ends of the credible interval are less probable. Models were adjusted for BMI and smoking. With the hierarchical regression analysis, the point estimates and their corresponding credible intervals were shifted toward each other (called “shrinkage” or “partial pooling”) [19]. This approach was used to address the issue of multiple comparisons, since we examined a large number of exposures [19]. Moreover, hierarchical regression can improve the accuracy of unstable estimates, when studying effects of multiple exposures with too few data, as in our study [19, 20]. For the hierarchical regression analysis, we specified the prior variance (τ) of 1.38, implying a prior expectation that 95% of the ORs would lie between 0.1 and 10. τ is a parameter used to control the strength of the common shrinkage of all the maximum-likelihood estimates toward their prior means. Although we chose a weak prior, it can be considered as a frequentist device to make the estimates stable, reducing their bias due to small sample size and, hence, increasing their accuracy [21, 22]. Hierarchical regression is described in more detail in the Additional file 1: Supplemental methods. All categorical variables were transformed into dummy variables and continuous variables were standardized (mean centered and rescaled by dividing by two standard deviations) [23].

In a secondary analysis, we estimated the association between reproductive and hormonal factors with pulmonary sarcoidosis (excluding extra-pulmonary cases) to restrict to a more homogenous sarcoidosis phenotype.

### Sensitivity analysis

We conducted several sensitivity analyses to test the robustness of the ORs from the main analysis. First, we used a prior τ of 0.125, implying a prior expectation that 95% of the ORs would lie between 0.5 and 2, to assess the sensitivity of our findings to the choice of a different prior. Second, we disregarded BMI and smoking from the main analysis to avoid adjustment for potential mediators as they were measured after the occurrence of some reproductive factors such as age at menarche and parity. Third, to address the low number of cases, we included the 10 sarcoidosis cases who had been diagnosed before the questionnaire date. Fourth, we performed the analysis among menopausal women since some reproductive factors such as age at menopause and HRT are related to menopause.

Data management and statistical analyses were performed using SAS software (version 9.4; SAS institute Inc., Cary, NC, USA).

## Results

A total of 32 cases and 124 controls were included in the study. The median age of cases and controls at questionnaire was 57 (cases range 40–72, controls range 39–72; Table 2). The median age of the cases at diagnosis was 69 years (range 54–82 years). Compared with controls, a larger percentage of cases reported naturally ceased menstruation (58.4% vs. 39.1%), using OC (68.8% vs. 58.3%) and HRT (54.2% vs. 37.0%), having shorter duration of OC use, and slightly shorter duration of local estrogen therapy (LET) use. In addition, a larger percentage of cases were non-smokers (84.4% vs. 71.7%) compared to controls. The majority of cases were pulmonary (91%) and 19% had Löfgren syndrome (see Additional file 1: Table C.1 for more detailed clinical characteristics).

**Table 2:** Characteristics of sarcoidosis cases and controls included from the Mammography Screening Project, 1995–2006

	Cases (n = 32)	Controls (n = 124)
Age at diagnosis (years), median (range)	69 (54–82)	
Age at questionnaire (years), median (range)	56.5 (40–72)	57 (39–72)
Age at menarche (years)	13.7 ± 1.3	13.3 ± 1.6
Age at menopause^a^ (years)	49.0 ± 6.9	49.9 ± 3.7
Menopausal status, N (%)		
Pre- or peri-menopausal	3 (9.4)	11 (8.9)
Post-menopausal	24 (75.0)	87 (70.2)
Unknown status	3 (9.4)	22 (17.7)
Missing	2 (6.2)	4 (3.2)
Natural menopause^¥,^^a^, N (%)		
No	2 (8.3)	13 (14.9)
Yes	14 (58.4)	35 (40.2)
Missing	8 (33.3)	39 (44.3)
Total menstrual lifespan (years)	30.4 ± 8.4	29.5 ± 7.8
Ever pregnant, N (%)		
Nulligravid	1 (3.1)	3 (2.4)
Gravid	29 (90.6)	110 (88.7)
Missing	2 (6.3)	11 (8.9)
Number of pregnancies	2.4 ± 1.1	2.6 ± 1.4
Age at first pregnancy^b^ (years)	25.9 ± 5.8	24.8 ± 4.6
Age at last pregnancy^b^ (years)	32.2 ± 6.1	31.5 ± 5.5
Years since last pregnancy^b^	25.8 ± 10.5	26.3 ± 9.6
OC use, N (%)		
Never	8 (25.0)	44 (35.5)
Ever	22 (68.8)	76 (61.3)
Missing	2 (6.2)	4 (3.2)
Duration of OC use^c^ (years)	4.1 ± 5.2	7.0 ± 6.5
Age at first OC use^c^ (years)	26.0 ± 6.5	25.3 ± 7.4
HRT use^a^, N (%)		
Never	10 (41.7)	51 (58.6)
Ever	13 (54.2)	33 (37.9)
Missing	1 (4.1)	3 (3.5)
Duration of HRT use^d^ (years)	2.6 ± 2.5	4.1 ± 4.1
Age at first HRT use^d^ (years)	50.3 ± 8.1	50.0 ± 5.2
LET use^a^, N (%)		
Never	17 (70.8)	55 (63.2)
Ever	6 (25.0)	26 (29.9)
Missing	1 (4.2)	6 (6.9)
Duration of LET use^e^ (years)	2.0 ± 1.6	2.9 ± 3.6
Body Mass Index (kg/m^2^)	26.8 ± 4.7	26.1 ± 4.0
Smoking, N (%)		
Non-smoker	27 (84.4)	89 (71.8)
smoker	3 (9.4)	31 (25.0)
Missing	2 (6.2)	4 (3.2)

Data are given as mean (± standard deviation), unless otherwise stated

*OC* oral contraceptive, *HRT* hormone replacement therapy, *LET* local estrogen therapy

^¥^Women who had undergone hysterectomy alone, ovariectomy alone or both hysterectomy and ovariectomy were considered as non-naturally menopausal

Numbers (n) in cases and controls are: ^a^among post-menopausal women, N = 111; ^b^among gravid women, N = 139; ^c^among ever OC users, N = 98; ^d^among ever HRT users and post-menopausal women, N = 46; ^e^among ever LET users and post-menopausal women, N = 32

The ORs of sarcoidosis were elevated for older age at menarche (OR 1.19: 95% CI 0.92–1.55), natural menopause vs. non-natural (OR 1.53: 95% CI 0.80–2.93), later age at first pregnancy (OR 1.11: 95% CI 0.76–1.63) and ever use of HRT (OR 1.40: 95% CI 0.76–2.59) (Tables 3, 4, 5). A lower odds of sarcoidosis were associated with older age at menopause (OR 0.90: 95% CI 0.52–1.55), longer duration of OC use (OR 0.70: 95% CI 0.45–1.07), longer duration of HRT use (OR 0.61: 95% CI 0.22–1.70), ever use of LET (OR 0.83: 95% CI 0.34–2.04) and longer duration of LET use (OR 0.78: 95% CI 0.21–2.81). However, the credible intervals cannot definitively rule out null associations (Tables 3, 4, 5). The ORs of sarcoidosis were close to 1 for the total menstrual lifespan, menopausal status (pre- or peri-menopausal vs. post-menopausal; pre- or peri-menopausal vs. unknown status), ever pregnant (gravid vs. nulligravid), number of pregnancies, age at and years since last pregnancy, ever OC use, age at first OC or HRT use (Tables 3, 4, 5). Results with different units (1-, 5- and 10-unit increments) for our continuous variables are presented in the Additional file 1: Table C.2. The ORs did not change notably when only those with pulmonary sarcoidosis were included (see Additional file 1: Table C.3).

**Table 3:** Association between proxies of endogenous hormone exposure and sarcoidosis in a matched case–control study of 156 women in the Mammography Screening Project, 1995–2006

	Cases (n = 32)*	Controls (n = 124)*	OR [95% CI]‡
Age at menarche (1-year increments)	29	116	1.19 [0.92–1.55]
Total menstrual lifespan (1-year increments)	24	89	1.02 [0.96–1.09]
Age at menopause^a^ (5-year increments)	22	81	0.90 [0.52–1.55]
Menopausal status			
Pre- or peri-menopausal	3	11	1 [ref]
Post-menopausal	24	87	1.05 [0.65–1.72]
Unknown status	3	22	0.54 [0.19–1.57]
Natural menopause^a, b^			
No	2	13	1 [ref]
Yes	14	35	1.53 [0.80–2.93]

*OR* odds ratio, *CI* credible interval

^*^Number of cases and controls with information on these variables

^‡^Odds ratios from hierarchical regression models, τ = 1.38 adjusted for smoking and body mass index

^a^Numbers (n) in cases and controls are among post-menopausal women, N = 111

^b^Women who had undergone hysterectomy alone, ovariectomy alone or both hysterectomy and ovariectomy were considered as non-naturally menopausal

**Table 4:** Association between pregnancy-related factors and sarcoidosis in a matched case–control study of 156 women in the Mammography Screening Project, 1995–2006

	Cases (n = 32)*	Controls (n = 124)*	OR [95% CI]‡
Ever pregnant			
Nulligravid	1	3	1 [ref]
Gravid	29	110	1.03 [0.67–1.60]
Number of pregnancies (1-pregnancy increments)	30	113	0.94 [0.68–1.29]
Age at first pregnancy^a^ (5-year increments)	28	109	1.11 [0.76–1.63]
Age at last pregnancy^a^ (5-year increments)	25	97	1.06 [0.73–1.55]
Years since last pregnancy^a^ (5-year increments)	25	97	0.99 [0.80–1.24]

OR odds ratio, *CI* credible interval

^*^Number of cases and controls with information on these variables

^‡^Odds ratios from hierarchical regression models, τ = 1.38 adjusted for smoking and body mass index

^a^Numbers (n) in cases and controls are among gravid women, N = 139

**Table 5:** Association between exogenous hormone exposure and sarcoidosis in a matched case–control study of 156 women in the Mammography Screening Project, 1995–2006

	Cases (n = 32)*	Controls (n = 124)*	OR [95% CI]‡
OC use			
Never	8	44	1 [ref]
Ever	22	76	1.08 [0.66–1.76]
Duration of OC use^a^ (5-year increments)	19	71	0.70 [0.45–1.07]
Age at first OC use^a^ (5-year increments)	22	74	1.06 [0.77–1.47]
HRT use^b^			
Never	10	51	1 [ref]
Ever	13	33	1.40 [0.76–2.59]
Duration of HRT use^c^ (5-year increments)	10	27	0.61 [0.22–1.70]
Age at first HRT use^c^ (5-year increments)	12	32	0.97 [0.57–1.65]
LET use^b^			
Never	17	55	1 [ref]
Ever	6	26	0.83 [0.34–2.04]
Duration of LET use^d^ (5-year increments)	4	24	0.78 [0.21–2.81]

*OR* odds ratio, *CI* credible interval, *OC* oral contraceptive, *HRT* hormone replacement therapy, *LET* local estrogen therapy

^*^Number of cases and controls with information on these variables

^‡^Odds ratios from hierarchical regression models, τ = 1.38 adjusted for smoking and body mass index

Numbers (n) in cases and controls are: ^a^among ever OC users, N = 98; ^b^among post-menopausal women, N = 111; ^c^among ever HRT users and post-menopausal women, N = 46; ^d^among ever LET users and post-menopausal women, N = 32

### Sensitivity analyses

Using a prior τ of 0.125 resulted in some attenuation of the ORs compared to the main analysis (see Additional file 1: Table C.4). In addition, the estimates did not change considerably when BMI and smoking were disregarded or when sarcoidosis cases diagnosed before questionnaire date were included (see Additional file 1: Tables C.5-C.6). Lastly, restricting to menopausal women resulted in the ORs increasing for age at menopause (from OR 0.90 to 1.31), and age at first HRT use (from OR 0.97 to 1.24; see Additional file 1: Table C.7).

## Discussion

The results from this nested case–control study in women in Västerbotten County indicate that older age at menopause, longer duration of OC, HRT or LET use and ever use of LET may be associated with reduced sarcoidosis risk. Older age at menarche, natural menopause vs. non-natural, older age at first pregnancy and ever use of HRT are likely to be associated with increased risk.

In sarcoidosis, granuloma formation is characterized by dominant expression of Th1 cytokines with low levels of expression of Th2 cytokines [24]. Th1 cytokines [interferon-γ, interleukin (IL)-2 and tumor necrosis factor-α] elicit phagocyte-dependent inflammation, while Th2 (IL-4, IL-10) inhibit the phagocyte function [25]. Evidence indicates that estrogens inhibit the production of Th1 proinflammatory cytokines, whereas they stimulate the production of Th2 anti-inflammatory cytokines [3, 26, 27]. Thus, estrogens may reduce sarcoidosis risk by improving the aberration of the Th1/Th2 balance.

Our study showed a decreased risk of sarcoidosis associated with higher age at menopause and younger age at menarche, which indicates a longer life-time exposure to estrogen. In addition, we found a lower risk of sarcoidosis associated with younger age at first pregnancy, which can be attributed to high estrogen concentrations during the intense hormonal changes involved in pregnancy [27, 28]. Our finding of a reduced sarcoidosis risk with later age at menopause is similar to results from a previous cohort study using data from the Black Women’s Health Study (BWHS) [6]. In contrast to our results, the BWHS found a decreased risk with later age at first birth and age at menarche was unrelated to sarcoidosis risk. A reason for these disparities could be due to differences in age at sarcoidosis diagnosis, since, in our study, the median age was 69 years, whereas in the BWHS it was 44 years. Furthermore, differences in estrogen levels between Black American women and Swedish women may have played a role. Some studies have suggested that estrogen levels are higher in black women compared to Caucasian women [29–31], while others have reported lower estrogen levels in black women [32, 33]. Moreover, we observed that naturally menopausal women had an increased sarcoidosis risk compared to non-naturally menopausal women (hysterectomy and/or ovariectomy). A potential explanation for this is that women with natural menopause (intact uterus) may have received a combination of estrogen and progesterone therapy or have not taken HRT, and are consequently exposed to lower levels of estrogen. In contrast, women undergoing hysterectomy and/or ovariectomy are likely to be exposed to higher levels of estrogen, since HRT with estrogen is generally prescribed after surgery [34].

Our results showed that a longer duration of OC, HRT, or LET, and ever use of LET may be related to a reduced risk of sarcoidosis, while the opposite was true for ever use of HRT. Moreover, no association was observed with ever OC use and age at first OC or HRT use. A potential explanation for these seemingly contradictory results might be the different types of OCs and HRT. There are two types of OCs; combined methods which contain both estrogen and progestin [35], and progestogen-only methods which contain only progesterone or synthetic progesterone-like substance (progestins) [36]. HRT can be either estrogen-only, or combined estrogen and progesterone/synthetic progesterone-like [34]. In line with our findings, the BWHS study in Black American women observed an elevated risk for ever HRT use and short-duration use. However, in analyses of OC use, the BWHS found no association [6]. A potential explanation may be the different combinations of OCs, both when it comes to compounds and doses between the two studies, as the prescription pattern may differ between Sweden and the United States. A review of OC use in the United States showed that almost 80 different formulations of OC were used [37], while in Europe, combined estrogen-progestogen contraceptives containing the progestogen levonorgestrel were used, as recommended by the European Medicines Agency [38]. Differences in the time period when women in the two studies received OCs can be another possible explanation. Women in the present study are older than the BWHS women and the estrogen dose of OCs has been altered over the past decades, using different types and combinations of hormones. It can also be possible that the women used different OCs and HRTs within the studies. Moreover, misclassification of self-reported exogenous estrogen variables from both studies and the small numbers of cases that used OC in our study may explain some of these discrepancies.

The validation of cases via review of medical records made our results robust to misclassification of sarcoidosis. We obtained prospectively collected data on multiple reproductive and hormonal factors from the Mammography Screening Project, which were used as proxies of estrogen exposure (endogenous and exogenous). These proxies were obtained prior to sarcoidosis diagnosis, minimizing the possibility of differential exposure misclassification (reverse causation bias). Lastly, the use of partial pooling in the hierarchical regression models yielded more efficient estimates by borrowing information from the different exposures while eliminating the need to adjust for multiplicity.

There are several limitations to our study. There may be some nondifferential misclassification of reproductive and hormonal factors due to the self-reported nature of our data. In addition, we could not distinguish between specific types of OC or HRT, as detailed data were not available in the MSP questionnaire; hence, their explicit effects on sarcoidosis were not possible to disentangle. Moreover, our study was limited by the small number of cases. To address this limitation, we used multilevel modelling and a weak prior to improve the precision and accuracy of unstable estimates. Due to small numbers of subjects with some exposures such as parity (few nulligravid women) and menopausal status (few pre- or peri-menopausal women), these exposures were difficult to study. Furthermore, there may be unmeasured confounding due to socioeconomic factors which have been found to be associated with pregnancy- [39] and hormone-related factors [40–42] as well as sarcoidosis severity [43].

Our results may only be generalizable to older onset sarcoidosis, since sarcoidosis diagnosed at a younger age may differ in terms of etiology (e.g. pathogenetic factors). Lastly, the generalizability of our findings may be limited to Caucasian women if the effect of reproductive factors on sarcoidosis risk is different between African American and Caucasian women due to differences in estrogen concentrations.

## Conclusions

Given the inconsistency and modest magnitude in our results across hormone-related factors, and that the 95% credible intervals included one, it still remains unclear if reproductive and hormonal factors are associated with sarcoidosis.

## Supplementary Information

**Additional file 1.** **Appendix A:** Mammography Survey. **Appendix B:** Supplemental Methods. **Appendix C:** Supplemental Tables.

## Data Availability

The datasets used and/or analysed for the current study cannot be shared as they are confidential but can be applied for from the Northern Sweden Health and Disease Study. Code for data cleaning and analysis is available from the corresponding author by request.

## Abbreviations

BMI: Body mass index
BWHS: Black Women’s Health Study
CI: Credible intervals
HRT: Hormone replacement therapy
ICD: International Classification of Diseases
IL: Interleukin
LET: Local estrogen therapy
MSP: Mammography Screening Project
OC: Oral contraceptives
OR: Odds ratio
Th: T-helper
