# Supplementary material for: Reproductive and hormonal risk factors for sarcoidosis: a nested case–control study
Source: BMC Pulm Med. 2022 Jan 24;22:43. doi: 10.1186/s12890-022-01834-1 (PMC8787880; doi:10.1186/s12890-022-01834-1)
Supplement: Supplementary file 1 — Additional file 1. Appendix A: Mammography Survey. Appendix B: Supplemental Methods. Appendix C: Supplemental Tables. [file 12890_2022_1834_MOESM1_ESM.docx]

**Supplement**

**Reproductive and hormonal risk factors for sarcoidosis: a nested case-control study**

Marina Dehara, Michael C Sachs, Susanna Kullberg, Johan Grunewald, Anders Blomberg, Elizabeth V Arkema

**Contents**

APPENDIX A: MAMMOGRAPHY SURVEY 2

APPENDIX B: SUPPLEMENTAL METHODS 5

Bayesian hierarchical regression analysis by data augmentation 5

Hierarchical regression 5

Data augmentation procedure 6

Posterior estimates and credible intervals calculations 10

References 11

APPENDIX C: SUPPLEMENTAL TABLES 12

Table C.1. 12

Table C.2. 14

Table C.3. 15

Table C.4. 16

Table C.5. 17

Table C.6. 18

Table C.7. 19

APPENDIX D: STROBE STATEMENT 20

## APPENDIX A: MAMMOGRAPHY SURVEY

**LÖRNR**

**PERSONNNUMER**………………………………………………………………………………………………………….………

**EFTERNAN**………………..………………………………………………………………………………………………..………….

**FÖRNAMN**………………………………………………………………………………………………………………….…………..

**HEMKOMMUN**………………………………………………………………………………………………………………………

**NATIONALITET**……………………………………………………..**DATUM**……………………………….................

**LÄNGD**……………………………………………………………….…...**VIKT**……………………………….......................

1. **Har någon i släkten haft** **Vem/Vilka (ange släktskap)**
2. Bröstcancer? ………………………………………………

1. Underlivscancer? Vilken typ? ……………………………..………………

Äggstockscancer? ……………………………..……………….

Livmodercancer? …………………………….………..……..

1. Magsäckscancer? …………………………….……………….
2. Tarmcancer? ……………………………………………..

1. Annan cancer? Vilken typ? ………………………………………………………………………
2. **Ålder vid första menstruation?** ………………………..…år
3. **Menstruationen har upphört** Nej Ja
4. **Datum/år för senaste menstruation** ………………………………
5. **Antal** **graviditeter**…………………….st Första graviditet……….……………….…årtal

Sista graviditet………………..………..…årtal

1. **Antal förlossningar**………………….st Första förlossning…….…….…….…..…årtal

Sista förlossning………………….…….…årtal

1. **Har du någon gång använt p-piller**

**inberäknat minipiller?** Nej Ja

1. **Hur länge har du använt**

**p-piller/minipiller?** ………………………..…år

1. **Hur gammal var du första gången du**

**använde p-piller/minipiller?** ………………………..…år

1. **Har du blivit rekommenderad av lättare**

**att sluta med p-piller/minipiller?** Nej Ja

1. **Har menstruationen någon gång - bort-**

**sett graviditet/amning - uteblivit mer**

**än 6 månader?** Nej Ja

1. **Under hur många månader var**

**menstruationen borta?** …………………….…mån

1. **Menstruationen upphörde i samband med**
   1. Matvägran
   2. Bantning
   3. Fysisk träning
   4. Upphörde naturligt
   5. Operation: Äggstockar borttagna

Livmoder borttagen

Livmoder- och äggstocksopererad

- 1. Annan orsak, vilken? .………………………………………………………………………………
  2. Vet ej

**Uppge dina Mediciner, även hormoner, de senaste 7 dagarna:**

………………………………………………………………………………………………………………………………………………

………………………………………………………………………………………………………………………………………………

1. **Har du kommit i övergångsåldern**

**(klimakteriet)?** Nej Ja, vid………...…års ålder

1. **Tar du nu eller har du tidigare tagit**

**hormontabletter mot övergångsbesvär?** Nej Ja Vet ej

1. **Hur gammal var du när du tog**

**hormontabletter mot övergångsbesvär**

**första gånger?** ………………………..…år

1. **Hur länge har du sammanlagt tagit**

**hormontabletter mot övergångsbesvär?** …………………….…mån

1. **Har du använt estrogen (tabletter eller**

**lokalbehandling) mot torra slemhinnor?** Nej Ja. Hur länge?..……mån

1. **Hur tycker du att ditt hälsotillstånd**

**varit det senaste äret**

1. Mycket gott
2. Ganska gott
3. Någorlunda
4. Tamligen dåligt
5. Dåligt

1. Sjukhusvårdad Nej Ja, anledning…………...…...

…………………………………………..

årtal……………………………………

1. Regelbunda läkarkontakter Nej Ja, anledning…………...…...

…………………………………………..

1. Bukopererad Nej Ja, anledning…………...…...

…………………………………………..

**Tack för din medverkan!**

More details about the variables used can be found here: <https://www.umu.se/en/biobank-research-unit/research/access-to-samples-and-data/access-to-nsdd/>

APPENDIX B: SUPPLEMENTAL METHODS

**Bayesian hierarchical regression analysis by data augmentation**

***Hierarchical regression***

Hierarchical regression can be thought of as a compromise between two extremes as described by Gelman *et al.* [1].

1. One extreme is full pooling, which assumes that the effects of reproductive factors simultaneously on sarcoidosis are the same across all reproductive factors (fixed effect). However, this approach does not account for possible correlation between reproductive factors, which can lead to false positive findings.

Full pooling assumes logit[P(Y = 1 | X, Z_i_, C)] = α + β × X + δ × C

where Y is a dichotomous variable representing the sarcoidosis status (Y=1: cases; Y=0: controls), X represents the exposure status of all the 19 reproductive factors combined, Z_i_ (i=1,..,19) represents the exposure status to the i^th^ reproductive factor, C is a vector of covariates included in the model (i.e. body mass index and smoking status), α is the intercept term, β is the regression coefficient corresponding to the combined 19 reproductive factors, and δ is the vector of regression coefficients corresponding to the covariates.

1. The other extreme is no pooling, which estimates the effect of each of the 19 reproductive factors separately on sarcoidosis risk (random effect).

No pooling assumes logit[P(Y = 1 | X, Z_i_, C)] = α + Σ_i_ (γ_i_ × X × Z_i_) + δ × C

where γ_i_ is the regression coefficient corresponding to the interaction between X and Z_i_, and γ_i_ ~ N(m, E).

1. Hierarchical regression is a compromise between full and no pooling, referred to as partial pooling. Partial pooling combines information from the fixed effect of the full pooling model and the random effects of the no pooling model.

Partial pooling assumes logit[P(Y = 1 | X, Z_i_)] = α + Σ_i_ X(β + γ_i_ × Z_i_) =

= α + β × X + (Σ_i_ γ_i_ × X × Z_i_) + δ × C

***Data augmentation procedure***

Before we performed the Bayesian hierarchical regression analysis, the following steps were taken to create the final dataset:

1. First, we re-structured our data by creating the G matrix. G is a 19 × 26 matrix which is structured with one row for each of the 19 reproductive factors. Each row is composed of 26 elements; the subject´s ID, sarcoidosis status (Y), the value of each reproductive factor (X), BMI, smoking, the total number of subjects represented per row in the data matrix G (M), the matched set identifier (caseset), followed by 19 elements (Z_i_) containing indicator variables for each reproductive factor.
2. Then, we created the J matrix. J is a 19 × 26 matrix, similar to the G matrix but instead of 19 Z_i_ columns, it has 19 W_i_ columns which represent the interaction between the value of each reproductive factor and the indicator variables for each reproductive factor.

We have provided an example showing a section of the matrix G and J for four reproductive factors in Table B.1 and B.2 (all data displayed here are made up for illustrative purposes).

| Table B.1. Section of the data matrix G for four reproductive factors | | | | | | | | | | |
| --- | --- | --- | --- | --- | --- | --- | --- | --- | --- | --- |
| ID | Y | X | Z_1_ | Z_2_ | Z_3_ | Z_4_ | BMI | smoking | M | Caseset |
| 1222 | 0 | 0.54 | 1 | 0 | 0 | 0 | 28.4 | 1 | 1 | 20030 |
| 1222 | 0 | 1.18 | 0 | 1 | 0 | 0 | 28.4 | 1 | 1 | 20030 |
| 1222 | 0 | -0.06 | 0 | 0 | 1 | 0 | 28.4 | 1 | 1 | 20030 |
| 1222 | 0 | 0.03 | 0 | 0 | 0 | 1 | 28.4 | 1 | 1 | 20030 |
| 1223 | 1 | 0.05 | 1 | 0 | 0 | 0 | 30 | 0 | 1 | 20031 |
| 1223 | 1 | 1.53 | 0 | 1 | 0 | 0 | 30 | 0 | 1 | 20031 |
| 1223 | 1 | 0.23 | 0 | 0 | 1 | 0 | 30 | 0 | 1 | 20031 |
| 1223 | 1 | -0.49 | 0 | 0 | 0 | 1 | 30 | 0 | 1 | 20031 |
| BMI; body mass index.  Y refers to sarcoidosis status (yes=1, no=0).  X refers to the value of the reproductive factor.  Zi refers to the indicator for each reproductive factor.  M is the total number of subjects represented per row in the data matrix G.  ID is the individual’s study identification number.  Caseset identifies the matched sets. | | | | | | | | | | |

| Table B.2. Section of the data matrix J for four reproductive factors | | | | | | | | | | |
| --- | --- | --- | --- | --- | --- | --- | --- | --- | --- | --- |
| ID | Y | X | W_1_ | W_2_ | W_3_ | W_4_ | BMI | smoking | M | Caseset |
| 1222 | 0 | 0.54 | 0.54 | 0 | 0 | 0 | 28.4 | 1 | 1 | 20030 |
| 1222 | 0 | 1.18 | 0 | 1.18 | 0 | 0 | 28.4 | 1 | 1 | 20030 |
| 1222 | 0 | -0.06 | 0 | 0 | -0.06 | 0 | 28.4 | 1 | 1 | 20030 |
| 1222 | 0 | 0.03 | 0 | 0 | 0 | 0.03 | 28.4 | 1 | 1 | 20030 |
| 1223 | 1 | 0.05 | 0.05 | 0 | 0 | 0 | 30 | 0 | 1 | 20031 |
| 1223 | 1 | 1.53 | 0 | 1.53 | 0 | 0 | 30 | 0 | 1 | 20031 |
| 1223 | 1 | 0.23 | 0 | 0 | 0.23 | 0 | 30 | 0 | 1 | 20031 |
| 1223 | 1 | -0.49 | 0 | 0 | 0 | -0.49 | 30 | 0 | 1 | 20031 |
| BMI; body mass index.  Y refers to sarcoidosis status (yes=1, no=0).  X refers to the value of the reproductive factor.  Zi refers to the indicator of which reproductive factor.  Wi is the interaction between X×Z_i._  M is the total number of subjects represented per row in the data matrix J.  ID is the individual’s study identification number.  Caseset identifies the matched sets. | | | | | | | | | | |

1. After creating the J matrix, we next created the prior data matrix. We used data augmentation priors for Bayesian analyses of conditional logistic regression described by Sullivan *et al.* [2], Greenland & Christensen 2001 [3], and Greenland 2007 [4]. For conditional logistic regression, the prior data table contained two pairs of records: one with an exposed case and an unexposed control and one with an unexposed case and an exposed control. Each record included the following:
2. *Y* is the number of cases represented by the prior data matrix. *Y* was used as a weight to increase the prior record contribution to the data and was calculated from the prior as *Y=(2×S^2^) – (3/2).* In the data matrix J, *Y* is 1 for all sarcoidosis cases and 0 for their matched controls. In PROC LOGISTIC, *Y* is the *“events”* in the *“events/trials”* model statement.
3. *M* is the total number of subjects represented by the prior data matrix and calculated as *2×Y* for a symmetric prior. In the data matrix J, *M* is equal to 1. In PROC LOGISTIC, *M* is the *“trials”* in the *“events/trials”* model statement.
4. *f* indicates a non-zero-centred prior, calculated as - *β_prior_*/S. However, since we have a zero-centred prior (*β_prior_=0*), the *f* column was omitted.
5. *W* is the variable for which the prior represents. *W* was calculated as *[1/sqrt(T)] / S* and all other regressors were equal to zero.
6. In the prior data, we used a matched set identifier, *caseset*, to indicate the pair, which is unique to each matched set. In the data matrix J, *caseset* identifies the matched sets. Using the *“strata”* statement the data are stratified by the variable *caseset*.

We used a weak prior variance (T) of 1.38 that places about 95% probability on the odds ratio e^β^ falling between 0.1 and 10. Although we chose a weak prior, it can still be considered as a frequentist device to make the estimates stable, decreasing their bias due to small sample size, sparse data and variance, and hence increasing their accuracy [5, 6]. We did not have better prior information for any specific reproductive factor compared to the others, hence, in each analysis, the same value for T was assigned to all reproductive factors. We calculated the β_prior_ and T based on Sullivan *et al*. [2]:

β_prior_ = ln(OR_prior_) = [ln(OR_ub_) + ln(OR_lb_)] / 2 = [ln(10) + ln(0.1)] / 2 = 2.3025851 -2.3025851 / 2 = 0

β_prior_ = ln(1)

T = [ln(10) – ln(0.1) / 2*1.96]^2^ = (2.3025851 + 2.3025851 /3.92)^2^ = (4.6051702/3.92)^2^ = 1.38

T= 1.38

| **2 record pairs** | **1 record pair**  **(collapsed prior pair)** |
| --- | --- |
| S=10 | S =10 |
| Y = (2×S^2^) – 3/2 = 2(100) -3/2 =  = 200-1.5 ≃ 198 | Y + Y = 396 |
| M = 2×Y = 198×2= 396 | M + M = 792 |
| W_1_ = [1/sqrt(T)] / S = 0.85/10 = 0.085 | W_1_ = 0.085 |
| W_0_ = 0/S = 0 | W_0_ = 0 |
| f = - *β_prior_*/S = 0/10 = 0 | f=0 |

The first half of Table B.3 shows a prior data table for a N(ln(1),1.38) prior. *Y* is the same for all four records in the prior for the coefficient *β*, so the two pairs (exposed case-unexposed control & unexposed case-exposed control) are identical and can thus be collapsed into a single pair. The second half of the table shows a single, collapsed prior pair (exposed & unexposed).

Table B.3. Prior records for conditional logistic regression, *N*(ln(1),1.38), *S*=10

|  | **Caseset** | **Y**  **(2×S^2^) – (3/2)** | **W_j_**  **[1/sqrt(T)] / S** | **M**  **2Y** |
| --- | --- | --- | --- | --- |
| Exposed case | 1 | 198 | 0.085 | 396 |
| Unexposed control | 1 | 198 | 0 | 396 |
| Unexposed case | 2 | 198 | 0 | 396 |
| Exposed control | 2 | 198 | 0.085 | 396 |
|  |  |  |  |  |
| Exposed | 1 | 396 | 0.085 | 792 |
| Unexposed | 1 | 396 | 0 | 792 |

Caseset = matched set identifier; S = scaling factor = 10;

We have provided an example showing a section of the prior data matrix for four reproductive factors in Table B.4 (all data displayed here are made up for illustrative purposes).

| Table B.4. Section of the prior data matrix | | | | | | | | | | |
| --- | --- | --- | --- | --- | --- | --- | --- | --- | --- | --- |
| ID | Y | X | W_1_ | W_2_ | W_3_ | W_4_ | BMI | smoking | M | Caseset |
| 1224 | 396 | 0 | 0.085 | 0 | 0 | 0 | 0 | 0 | 792 | 20032 |
| 1225 | 396 | 0 | 0 | 0 | 0 | 0 | 0 | 0 | 792 | 20032 |
| 1226 | 396 | 0 | 0 | 0.085 | 0 | 0 | 0 | 0 | 792 | 20033 |
| 1227 | 396 | 0 | 0 | 0 | 0 | 0 | 0 | 0 | 792 | 20033 |
| 1228 | 396 | 0 | 0 | 0 | 0.085 | 0 | 0 | 0 | 792 | 20034 |
| 1229 | 396 | 0 | 0 | 0 | 0 | 0 | 0 | 0 | 792 | 20034 |
| 1230 | 396 | 0 | 0 | 0 | 0 | 0.085 | 0 | 0 | 792 | 20035 |
| 1231 | 396 | 0 | 0 | 0 | 0 | 0 | 0 | 0 | 792 | 20035 |
| BMI; body mass index.  Y is the number of cases; a weight to increase the prior record contribution to the data.  M is the total number of subjects in the record pair (exposed & unexposed).  Caseset is the matched set identifier.  W is the variable for which the prior represents, calculated as [1/sqrt(T)] / S=0.085 and all other regressors are zero.  Confounders (BMI & smoking) and X are zero.  ID is the individual’s study identification number. | | | | | | | | | | |

1. The final dataset was created by adding the prior data matrix to the data matrix J.

***Posterior estimates and credible intervals calculations***

Partial pooling equation: logit[P(Y = 1 | X, Z_i_)] = α + β × X + (Σ_i_ γ_i_ × X × Z_i_) + δ × C =

= α + β × X + (Σ_i_ γ_i_ × W_i_) + δ × C

The posterior estimates were calculated by taking the sum of estimates of the fixed (β) and random effects (γ_i_ = γ_1_,…, γ_19_).

The credible intervals (CI) were calculated from the standard error (*se*) of the sum of the estimated fixed and random effects, specifically:

1. First, the variance (*var*) of the sum was computed:

*var*(β + γ_i_) = *var*(β) + *var*(γ_i_) + 2**cov*(β, γ_i_) is the variance of β and γ_i_, where:

- - *var*(β) = *se*(β)^2^ is the variance of β.
  - *var*(γ_i_) = *se*(γ_i_)^2^ is the variance of γ_i_.
  - *cov*(β, γ_i_) is the covariance of β and γ_i_.

1. Then, the standard error was computed by taking the square root of the variance of the sum: *se* = √ *var*

3. Lastly, the credible intervals for the odds ratios were calculated as CI=exp(estimate +/-1.96×*se).*

**References**

1. Gelman A, Hill J, Yajima M. Why we (usually) don't have to worry about multiple comparisons. J Res Educ Eff. 2012;5(2):189-211. <https://doi.org/10.1080/19345747.2011.618213>
2. Sullivan SG, Greenland S. Bayesian regression in SAS software. Int J Epidemiol. 2013;42(1):308-17. <https://doi.org/10.1093/ije/dys213>
3. Greenland S, Christensen R. Data augmentation priors for Bayesian and semi-Bayes analyses of conditional-logistic and proportional-hazards regression. Stat Med. 2001;20(16):2421-8. <https://doi.org/10.1002/sim.902>
4. Greenland S. Bayesian perspectives for epidemiological research. II. Regression analysis. Int J Epidemiol. 2007;36(1):195-202. <https://doi.org/10.1093/ije/dyl289>
5. Greenland S, Schwartzbaum JA, Finkle WD. Problems due to small samples and sparse data in conditional logistic regression analysis. Am J Epidemiol. 2000;151(5):531-9. <https://doi.org/10.1093/oxfordjournals.aje.a010240>
6. Greenland S. Small-sample bias and corrections for conditional maximum-likelihood odds-ratio estimators. Biostatistics. 2000;1(1);113-22. <https://doi.org/10.1093/biostatistics/1.1.113>

APPENDIX C: SUPPLEMENTAL TABLES

| Table C.1. Clinical characteristics of sarcoidosis cases included in the study, (n=32) | |
| --- | --- |
| **Patient characteristic** | **N=32** |
| **Age at diagnosis, years, mean ± SD** | 69 (±8) |
| **First diagnosis received in respiratory clinic** | 94% |
| **Symptom onset before first visit** |  |
| Days | 6% |
| Months | 53% |
| Years | 32% |
| Missing | 9% |
| **Löfgren syndrome** | 19% |
| **Disease type** |  |
| Pulmonary | 91% |
| Extra-pulmonary | 3% |
| Both pulmonary and extra-pulmonary | 6% |
| **Clinical symptoms compatible with sarcoidosis** |  |
| No, not compatible | 3% |
| No symptoms | 9% |
| Yes, compatible | 32% |
| Unclear/Missing | 56% |
| **Chest X-ray compatible** |  |
| No, not compatible | 13% |
| Not performed initially | 28% |
| Yes, compatible | 22% |
| Unclear/Missing | 37% |
| **Scadding stage** (in those with a compatible chest X-ray) |  |
| 0 | 9% |
| 1 | 3% |
| 2 | 13% |
| 3 | 3% |
| Missing | 72% |
| **Other radiographic imaging performed** |  |
| CT | 72% |
| PET-CT | 19% |
| Only chest X-Ray | 9% |
| **Biopsy performed** | 75% |
| Of those with biopsy, % positive | 50% |
| **EBUS-TBNA performed** | 53% |
| **BAL performed** | 60% |
| Of those with BAL, |  |
| Lymphocytes >25% of total cell count | 37% |
| CD4/CD8 ratio >3.5 | 58% |
| **Serum angiotensin converting enzyme > upper limit of normal** |  |
| Yes | 31% |
| No | 60% |
| Not measured | 9% |
| **Serum calcium > upper limit of normal** |  |
| Yes | 12% |
| No | 72% |
| Not measured | 16% |
| **Received medication at initial diagnosis** | 19% |
| **Reviewing physician’s impression** |  |
| Definite | 81% |
| Probable but cannot say with 100% certainty | 19% |
| BAL; bronchoalveolar lavage, EBUS-TBNA; endobronchial ultrasound-guided transbronchial needle aspiration |  |

| Table C.2. Association between reproductive and hormonal factors with sarcoidosis in a matched case-control study of 156 women in the Mammography Screening Project, 1995-2006, using 1-, 5- and 10-unit increments. | | | |
| --- | --- | --- | --- |
|  | **1-unit increments** | **5-unit increments** | **10-unit increments** |
|  | OR [95% CI] | OR [95% CI] | OR [95% CI] |
| Age at menarche, years | 1.19 [0.92-1.55] | 2.43 [0.67-8.88] | 5.91 [0.44-78.84] |
| Total menstrual lifespan, years | 1.02 [0.96-1.09] | 1.11 [0.83-1.50] | 1.25 [0.69-2.26] |
| Age at menopause, years | 0.97 [0.88-1.09] | 0.90 [0.52-1.55] | 0.81 [0.27-2.39] |
| Number of pregnancies | 0.94 [0.68-1.29] | 0.72 [0.15-3.52] | 0.52 [0.02-12.39] |
| Age of first pregnancy, years | 1.02 [0.95-1.10] | 1.11 [0.76-1.63] | 1.23 [0.57-2.67] |
| Age of last pregnancy, years | 1.01 [0.94-1.09] | 1.06 [0.73-1.55] | 1.13 [0.53-2.39] |
| Years since last pregnancy | 1.00 [0.96-1.04] | 0.99 [0.80-1.24] | 0.99 [0.64-1.54] |
| Duration of OC use, years | 0.93 [0.85-1.01] | 0.70 [0.45-1.07] | 0.49 [0.20-1.16] |
| Age at first OC use, years | 1.01 [0.95-1.08] | 1.06 [0.77-1.47] | 1.13 [0.59-2.17] |
| Duration of HRT use, years | 0.90 [0.74-1.11] | 0.61 [0.22-1.70] | 0.37 [0.05-2.89] |
| Age at first HRT use, years | 0.99 [0.89-1.10] | 0.97 [0.57-1.65] | 0.94 [0.32-2.72] |
| Duration of LET, years | 0.95 [0.73-1.23] | 0.78 [0.21-2.81] | 0.60 [0.05-7.91] |
| OC; oral contraceptives, HRT; hormone replacement therapy, LET; local estrogen therapy | | | |

| Table C.3. Association between reproductive and hormonal factors with pulmonary sarcoidosis in a matched case-control study of 143 women in the Mammography Screening Project, 1995-2006. | | | | |
| --- | --- | --- | --- | --- |
|  | **Cases* (n=29)** | **Controls* (n=114)** | | **OR[95% CI]**‡ |
| Age at menarche (1-year increments) | 27 | | 108 | 1.16 [0.89-1.52] |
| Total menstrual lifespan (1-year increments) | 22 | | 81 | 1.01 [0.95-1.08] |
| Age at menopause^a^ (5-year increments) | 20 | | 73 | 0.69 [0.39-1.23] |
| Menopausal status |  | | |  |
| pre- or peri-menopausal | 3 | | 11 | 1 [ref] |
| post-menopausal | 22 | | 79 | 1.05 [0.63-1.76] |
| unknown status | 3 | | 22 | 0.54 [0.19-1.56] |
| Natural menopause^±, a^ |  | | |  |
| no | 2 | | 12 | 1 [ref] |
| yes | 14 | | 29 | 1.86 [0.94-3.66] |
| Ever pregnant |  | | |  |
| nulligravid | 1 | | 3 | 1 [ref] |
| gravid | 27 | | 103 | 1.02 [0.65-1.61] |
| Number of pregnancies (1-pregnancy increments) | 28 | | 106 | 0.97 [0.70-1.35] |
| Age of first pregnancy^b^ (5-year increments) | 26 | | 102 | 1.09 [0.73-1.62] |
| Age of last pregnancy^b^ (5-year increments) | 23 | | 90 | 1.09 [0.73-1.61] |
| Years since last pregnancy^b^ (5-year increments) | 23 | | 90 | 0.98 [0.77-1.23] |
| OC use |  | | |  |
| never | 7 | | 37 | 1 [ref] |
| ever | 21 | | 75 | 1.05 [0.63-1.73] |
| Duration of OC use^c^ (5-year increments) | 18 | | 70 | 0.70 [0.45-1.09] |
| Age at first OC use^c^ (5-year increments) | 21 | | 73 | 1.06 [0.75-1.51] |
| HRT use^a^ |  | | |  |
| never | 9 | | 46 | 1 [ref] |
| ever | 12 | | 31 | 1.39 [0.74-2.62] |
| Duration of HRT use^d^ (5-year increments) | 10 | | 25 | 0.58 [0.20-1.69] |
| Age at first HRT use^d^ (5-year increments) | 11 | | 30 | 0.72 [0.39-1.35] |
| LET use^a^ |  | | |  |
| never | 16 | | 53 | 1 [ref] |
| ever | 5 | | 20 | 0.86 [0.32-2.34] |
| Duration of LET use^e^ (5-year increments) | 3 | | 18 | 0.70 [0.18-2.69] |
| OR; Odds Ratio, CI; Credible Interval, OC; oral contraceptive, HRT; hormone replacement therapy, LET; local estrogen therapy.  *Number of cases and controls with information on these variables.  ‡Odds ratios from hierarchical regression models, Τ=1.38 adjusted for smoking and body mass index.  ^±^Women who had undergone hysterectomy alone, ovariectomy alone or both hysterectomy and ovariectomy were considered as non-naturally menopausal.  Numbers (n) in cases and controls are: ^a^among post-menopausal women, N=101; ^b^among gravid women, N=130; ^c^among ever OC users, N=96; ^d^among ever HRT users and post-menopausal women, N=43; ^e^among ever LET users and post-menopausal women, N=25. | | | | |

| Table C.4. Association between reproductive and hormonal factors with sarcoidosis in a matched case-control study of 156 women in the Mammography Screening Project, 1995-2006. Odds ratios of sarcoidosis and 95% CIs obtained using hierarchical regression models with a prior variance of 0.125. | | | | |
| --- | --- | --- | --- | --- |
|  | **Cases (n=32)*** | **Controls (n=124)*** | | **OR[95% CI]**‡ |
| Age at menarche (1-year increments) | 29 | | 116 | 1.09 [0.91-1.30] |
| Total menstrual lifespan (1-year increments) | 24 | | 89 | 1.01 [0.97-1.05] |
| Age at menopause^a^ (5-year increments) | 22 | | 81 | 0.97 [0.68-1.39] |
| Menopausal status |  | | |  |
| pre- or peri-menopausal | 3 | | 11 | 1 [ref] |
| post-menopausal | 24 | | 87 | 1.04 [0.68-1.58] |
| unknown status | 3 | | 22 | 0.82 [0.45-1.51] |
| Natural menopause^±, a^ |  | | |  |
| no | 2 | | 13 | 1 [ref] |
| yes | 14 | | 35 | 1.28 [0.76-2.14] |
| Ever pregnant |  | | |  |
| nulligravid | 1 | | 3 | 1 [ref] |
| gravid | 29 | | 110 | 1.03 [0.70-1.51] |
| Number of pregnancies (1-pregnancy increments) | 30 | | 113 | 0.98 [0.80-1.21] |
| Age of first pregnancy^b^ (5-year increments) | 28 | | 109 | 1.06 [0.80-1.42] |
| Age of last pregnancy^b^ (5-year increments) | 25 | | 97 | 1.04 [0.80-1.35] |
| Years since last pregnancy^b^ (5-year increments) | 25 | | 97 | 1.00 [0.86-1.16] |
| OC use |  | | |  |
| never | 8 | | 44 | 1 [ref] |
| ever | 22 | | 76 | 1.06 [0.70-1.61] |
| Duration of OC use^c^ (5-year increments) | 19 | | 71 | 0.89 [0.70-1.14] |
| Age at first OC use^c^ (5-year increments) | 22 | | 74 | 1.03 [0.83-1.27] |
| HRT use^a^ |  | | |  |
| never | 10 | | 51 | 1 [ref] |
| ever | 13 | | 33 | 1.23 [0.75-2.02] |
| Duration of HRT use^d^ (5-year increments) | 10 | | 27 | 0.91 [0.56-1.49] |
| Age at first HRT use^d^ (5-year increments) | 12 | | 32 | 1.00 [0.74-1.35] |
| LET use^a^ |  | | |  |
| never | 17 | | 55 | 1 [ref] |
| ever | 6 | | 26 | 0.94 [0.52-1.68] |
| Duration of LET use^e^ (5-year increments) | 4 | | 24 | 0.98 [0.56-1.69] |
| OR; Odds Ratio, CI; Credible Interval, OC; oral contraceptive, HRT; hormone replacement therapy, LET; local estrogen therapy.  *Number of cases and controls with information on these variables.  ‡Odds ratios from hierarchical regression models, Τ=0.125 adjusted for smoking and body mass index.  ^±^Women who had undergone hysterectomy alone, ovariectomy alone or both hysterectomy and ovariectomy were considered as non-naturally menopausal  Numbers (n) in cases and controls are: ^a^among post-menopausal women, N=111; ^b^among gravid women, N=139; ^c^among ever OC users, N=98; ^d^among ever HRT users and post-menopausal women, N=46; ^e^among ever LET users and post-menopausal women, N=32. | | | | |

| Table C.5. Association between reproductive and hormonal factors with sarcoidosis in a matched case-control study of 156 women in the Mammography Screening Project, 1995-2006. Odds ratios of sarcoidosis and 95% CIs unadjusted for body mass index and smoking. | | | |
| --- | --- | --- | --- |
|  | **Cases (n=32)*** | **Controls (n=124)*** | **OR[95% CI]‡** |
| Age at menarche (1-year increments) | 29 | 116 | 1.18 [0.92-1.50] |
| Total menstrual lifespan (1-year increments) | 24 | 89 | 1.01 [0.96-1.07] |
| Age at menopause^a^ (5-year increments) | 22 | 81 | 0.83 [0.51-1.35] |
| Menopausal status |  | |  |
| pre- or peri-menopausal | 3 | 11 | 1 [ref] |
| post-menopausal | 24 | 87 | 1.10 [0.70-1.75] |
| unknown status | 3 | 22 | 0.57 [0.20-1.63] |
| Natural menopause^±, a^ |  | |  |
| no | 2 | 13 | 1 [ref] |
| yes | 14 | 35 | 1.47 [0.80-2.71] |
| Ever pregnant |  | |  |
| nulligravid | 1 | 3 | 1 [ref] |
| gravid | 29 | 110 | 1.04 [0.68-1.58] |
| Number of pregnancies (1-pregnancy increments) | 30 | 113 | 0.86 [0.63-1.17] |
| Age of first pregnancy^b^ (5-year increments) | 28 | 109 | 1.20 [0.82-1.76] |
| Age of last pregnancy^b^ (5-year increments) | 25 | 97 | 1.10 [0.76-1.58] |
| Years since last pregnancy^b^ (5-year increments) | 25 | 97 | 0.99 [0.80-1.23] |
| OC use |  | |  |
| never | 8 | 44 | 1 [ref] |
| ever | 22 | 76 | 1.08 [0.67-1.75] |
| Duration of OC use^c^ (5-year increments) | 19 | 71 | 0.70 [0.46-1.08] |
| Age at first OC use^c^ (5-year increments) | 22 | 74 | 1.06 [0.77-1.45] |
| HRT use^a^ |  | |  |
| never | 10 | 51 | 1 [ref] |
| ever | 13 | 33 | 1.39 [0.77-2.51] |
| Duration of HRT use^d^ (5-year increments) | 10 | 27 | 0.53 [0.20-1.45] |
| Age at first HRT use^d^ (5-year increments) | 12 | 32 | 1.06 [0.63-1.80] |
| LET use^a^ |  | |  |
| never | 17 | 55 | 1 [ref] |
| ever | 6 | 26 | 0.96 [0.41-2.22] |
| Duration of LET use^e^ (5-year increments) | 4 | 24 | 0.74 [0.22-2.52] |
| OR; Odds Ratio, CI; Credible Interval, OC; oral contraceptive, HRT; hormone replacement therapy, LET; local estrogen therapy.  *Number of cases and controls with information on these variables.  ‡Odds ratios from hierarchical regression models, Τ=1.38.  ^±^Women who had undergone hysterectomy alone, ovariectomy alone or both hysterectomy and ovariectomy were considered as non-naturally menopausal  Numbers (n) in cases and controls are: ^a^among post-menopausal women, N=111; ^b^among gravid women, N=139; ^c^among ever OC users, N=98; ^d^among ever HRT users and post-menopausal women, N=46; ^e^among ever LET users and post-menopausal women, N=32. | | | |

| Table C.6. Association between reproductive and hormonal factors with sarcoidosis cases in a matched case-control study of 209 women in the Mammography Screening Project, 1995-2006. Cases who were diagnosed before the questionnaire date (n=10) were also included in this sensitivity analysis. | | | | |
| --- | --- | --- | --- | --- |
|  | **Cases (n=42)*** | | **Controls (n=167)*** | **OR[95% CI]‡** |
| Age at menarche (1-year increments) | 39 | 154 | | 1.14 [0.92-1.43] |
| Total menstrual lifespan (1-year increments) | 31 | 122 | | 1.01 [0.96-1.07] |
| Age at menopause^a^ (5-year increments) | 27 | 108 | | 0.81 [0.49-1.33] |
| Menopausal status |  | | |  |
| pre- or peri-menopausal | 5 | 17 | | 1 [ref] |
| post-menopausal | 30 | 121 | | 0.98 [0.63-1.51] |
| unknown status | 5 | 25 | | 0.77 [0.31-1.92] |
| Natural menopause^±, a^ |  | | |  |
| no | 3 | 23 | | 1 [ref] |
| yes | 18 | 48 | | 1.50 [0.85-2.67] |
| Ever pregnant |  | | |  |
| nulligravid | 1 | 6 | | 1 [ref] |
| gravid | 39 | 148 | | 1.07 [0.73-1.56] |
| Number of pregnancies (1-pregnancy increments) | 40 | 154 | | 0.93 [0.70-1.23] |
| Age of first pregnancy^b^ (5-year increments) | 38 | 145 | | 1.17 [0.82-1.66] |
| Age of last pregnancy^b^ (5-year increments) | 33 | 129 | | 1.07 [0.76-1.52] |
| Years since last pregnancy^b^ (5-year increments) | 33 | 129 | | 0.97 [0.80-1.19] |
| OC use |  | | |  |
| never | 11 | 59 | | 1 [ref] |
| ever | 29 | 102 | | 1.11 [0.72-1.71] |
| Duration of OC use^c^ (5-year increments) | 26 | 95 | | 0.72 [0.49-1.08] |
| Age at first OC use^c^ (5-year increments) | 29 | 99 | | 1.05 [0.78-1.42] |
| HRT use^a^ |  | | |  |
| never | 14 | 71 | | 1 [ref] |
| ever | 14 | 47 | | 1.11 [0.63-1.96] |
| Duration of HRT use^d^ (5-year increments) | 11 | 38 | | 0.91 [0.37-2.23] |
| Age at first HRT use^d^ (5-year increments) | 13 | 46 | | 1.06 [0.64-1.75] |
| LET use^a^ |  | | |  |
| never | 21 | 80 | | 1 [ref] |
| ever | 7 | 32 | | 0.76 [0.33-1.74] |
| Duration of LET use^e^ (5-year increments) | 5 | 29 | | 0.79 [0.23-2.72] |
| OR; Odds Ratio, CI; Credible Interval, OC; oral contraceptive, HRT; hormone replacement therapy, LET; local estrogen therapy.  *Number of cases and controls with information on these variables.  ‡Odds ratios from hierarchical regression models, Τ=1.38 adjusted for smoking and body mass index.  ^±^Women who had undergone hysterectomy alone, ovariectomy alone or both hysterectomy and ovariectomy were considered as non-naturally menopausal  Numbers (n) in cases and controls are: ^a^among post-menopausal women, N=151; ^b^among gravid women, N=187; ^c^among ever OC users, N=131; ^d^among ever HRT users and post-menopausal women, N=61; ^e^among ever LET users and post-menopausal women, N=39. | | | | |

| Table C.7. Association between reproductive and hormonal factors with sarcoidosis among menopausal women only in a matched case-control study of 104 women in the Mammography Screening Project, 1995-2006. | | | | | |
| --- | --- | --- | --- | --- | --- |
|  | **Cases (n=24)*** | | **Controls (n=80)*** | | **OR[95% CI]‡** |
| Age at menarche (1-year increments) | 23 | | 79 | | 1.43 [1.03-1.99] |
| Total menstrual lifespan (1-year increments) | 21 | | 73 | | 1.05 [0.98-1.13] |
| Age at menopause (5-year increments) | 22 | | 74 | | 1.31 [0.74-2.30] |
| Natural menopause^a^ |  | | | |  |
| no | 2 | | 11 | | 1 [ref] |
| yes | 14 | | 32 | | 1.60 [0.81-3.15] |
| Ever pregnant |  | | | |  |
| nulligravid | 1 | | 2 | | 1 [ref] |
| gravid | 23 | | 74 | | 1.07 [0.64-1.80] |
| Number of pregnancies (1-pregnancy increments) | 24 | | 76 | | 0.86 [0.60-1.24] |
| Age of first pregnancy^a^ (5-year increments) | 22 | | 74 | | 1.17 [0.75-1.82] |
| Age of last pregnancy^a^ (5-year increments) | 20 | | 67 | | 1.04 [0.65-1.65] |
| Years since last pregnancy^a^ (5-year increments) | 20 | | 67 | | 1.03 [0.76-1.39] |
| OC use |  | | |  | |
| never | 7 | 33 | | 1 [ref] | |
| ever | 17 | 47 | | 1.10 [0.61-2.02] | |
| Duration of OC use^b^ (5-year increments) | 15 | 43 | | 0.58 [0.36-0.96] | |
| Age at first OC use^b^ (5-year increments) | 17 | 46 | | 1.07 [0.71-1.61] | |
| HRT use |  | | |  | |
| never | 10 | 45 | | 1 [ref] | |
| ever | 13 | 32 | | 1.32 [0.67-2.63] | |
| Duration of HRT use^c^ (5-year increments) | 10 | 26 | | 0.74 [0.27-2.04] | |
| Age at first HRT use^c^ (5-year increments) | 12 | 31 | | 1.24 [0.71-2.16] | |
| LET use |  | | |  | |
| never | 17 | 48 | | 1 [ref] | |
| ever | 6 | 26 | | 0.83 [0.34-2.06] | |
| Duration of LET use^d^ (5-year increments) | 4 | 24 | | 0.85 [0.23-3.12] | |
| OR; Odds Ratio, CI; Credible Interval, OC; oral contraceptive, HRT; hormone replacement therapy, LET; local estrogen therapy.  *Number of cases and controls with information on these variables.  ‡Odds ratios from hierarchical regression models, Τ=1.38 adjusted for smoking and body mass index.  ^a^Women who had undergone hysterectomy alone, ovariectomy alone or both hysterectomy and ovariectomy were considered as non-naturally menopausal  Numbers (n) in cases and controls are: ^a^among gravid women, N=97; ^b^among ever OC users, N=64; ^c^among ever HRT users, N=45; ^d^among ever LET users, N=32. | | | | | |

APPENDIX D: STROBE STATEMENT

STROBE Statement—Checklist of items that should be included in reports of ***case-control studies***

|  | Item No | Recommendation | | | Section/Paragraph | |
| --- | --- | --- | --- | --- | --- | --- |
| **Title and abstract** | 1 | (*a*) Indicate the study’s design with a commonly used term in the title or the abstract | | | Title | |
|  |  | (*b*) Provide in the abstract an informative and balanced summary of what was done and what was found | | | Abstract | |
| Introduction | | | | |  | |
| Background/rationale | 2 | Explain the scientific background and rationale for the investigation being reported | | | Background, paragraph 1-2 | |
| Objectives | 3 | State specific objectives, including any prespecified hypotheses | | | Background, paragraph 3 | |
| Methods | | | | |  | |
| Study design | 4 | Present key elements of study design early in the paper | | | Methods, Study population | |
| Setting | 5 | Describe the setting, locations, and relevant dates, including periods of recruitment, exposure, follow-up, and data collection | | | Methods, Study population | |
| Participants | 6 | (*a*) Give the eligibility criteria, and the sources and methods of case ascertainment and control selection. Give the rationale for the choice of cases and controls | | | Methods, Identification of cases and controls | |
|  |  | (*b*) For matched studies, give matching criteria and the number of controls per case | | | Methods, Identification of cases and controls | |
| Variables | 7 | Clearly define all outcomes, exposures, predictors, potential confounders, and effect modifiers. Give diagnostic criteria, if applicable | | | Methods, Identification of cases and controls, Exposures: reproductive and hormonal factors, and Other variables section | |
| Data sources/ measurement | 8* | For each variable of interest, give sources of data and details of methods of assessment (measurement). Describe comparability of assessment methods if there is more than one group | | | Methods, Study population, and Identification of cases and controls | |
| Continued on next page | | |  |  | |  |

| Bias | 9 | Describe any efforts to address potential sources of bias | | | Discussion, paragraph 5 | |
| --- | --- | --- | --- | --- | --- | --- |
| Study size | 10 | Explain how the study size was arrived at | | | Methods, Study population, and Identification of cases and controls | |
| Quantitative variables | 11 | Explain how quantitative variables were handled in the analyses. If applicable, describe which groupings were chosen and why | | | Methods, Exposures: reproductive and hormonal factors | |
| Statistical methods | 12 | (*a*) Describe all statistical methods, including those used to control for confounding | | | Methods, Statistical analysis | |
|  |  | (*b*) Describe any methods used to examine subgroups and interactions | | | N/A | |
|  |  | (*c*) Explain how missing data were addressed | | | Very few missing data, see Table 2 | |
|  |  | (*d*) If applicable, explain how matching of cases and controls was addressed | | | N/A | |
|  |  | (*e*) Describe any sensitivity analyses | | | Methods, Sensitivity analysis | |
| Results | | | | |  | |
| Participants | 13* | (a) Report numbers of individuals at each stage of study—eg numbers potentially eligible, examined for eligibility, confirmed eligible, included in the study, completing follow-up, and analysed | | | Methods, Study population, and Identification of cases and controls | |
|  |  | (b) Give reasons for non-participation at each stage | | | Methods, Study population, and Identification of cases and controls | |
|  |  | (c) Consider use of a flow diagram | | |  | |
| Descriptive data | 14* | (a) Give characteristics of study participants (eg demographic, clinical, social) and information on exposures and potential confounders | | | Table 2, see also Additional file 1, Table C.1 | |
|  |  | (b) Indicate number of participants with missing data for each variable of interest | | | Table 2 | |
| Continued on next page | | |  |  | |  |

| Outcome data | 15* | Report numbers in each exposure category, or summary measures of exposure | Table 2 |
| --- | --- | --- | --- |
| Main results | 16 | (*a*) Give unadjusted estimates and, if applicable, confounder-adjusted estimates and their precision (eg, 95% confidence interval). Make clear which confounders were adjusted for and why they were included | Table 3-5 |
|  |  | (*b*) Report category boundaries when continuous variables were categorized | N/A |
|  |  | (*c*) If relevant, consider translating estimates of relative risk into absolute risk for a meaningful time period | N/A |
| Other analyses | 17 | Report other analyses done—eg analyses of subgroups and interactions, and sensitivity analyses | Results, Sensitivity analysis  See also Additional file 1, APPENDIX C: SUPPLEMENTAL TABLES |
| Discussion | | |  |
| Key results | 18 | Summarise key results with reference to study objectives | Discussion, paragraph 1 |
| Limitations | 19 | Discuss limitations of the study, taking into account sources of potential bias or imprecision. Discuss both direction and magnitude of any potential bias | Discussion, paragraph 6 |
| Interpretation | 20 | Give a cautious overall interpretation of results considering objectives, limitations, multiplicity of analyses, results from similar studies, and other relevant evidence | Discussion, paragraph 2-4 |
| Generalisability | 21 | Discuss the generalisability (external validity) of the study results | Discussion, paragraph 7 |
| Other information | | |  |
| Funding | 22 | Give the source of funding and the role of the funders for the present study and, if applicable, for the original study on which the present article is based | Described in funding section |

*Give information separately for cases and controls.

**Note:** An Explanation and Elaboration article discusses each checklist item and gives methodological background and published examples of transparent reporting. The STROBE checklist is best used in conjunction with this article (freely available on the Web sites of PLoS Medicine at http://www.plosmedicine.org/, Annals of Internal Medicine at http://www.annals.org/, and Epidemiology at http://www.epidem.com/). Information on the STROBE Initiative is available at http://www.strobe-statement.org.
